# Is the Osmolal Concentration of Ethanol Greater Than Its Molar Concentration?

**DOI:** 10.3389/fmed.2019.00306

**Published:** 2020-01-08

**Authors:** Minhtri K. Nguyen, Lu Song, Liyo Kao, Kevin Tong, Maria J. De La Cruz, Giancarlo Rodriguez, Minh-Kevin Nguyen, Dai-Scott Nguyen, Ira Kurtz

**Affiliations:** David Geffen School of Medicine at UCLA, Los Angeles, CA, United States

**Keywords:** ethanol, osmolal gap, osmolality, methanol, ethylene glycol

## Abstract

**Background:** Recent data suggested that the osmolal gap attributed to ethanol as determined by the difference between *measured* serum osmolality and *calculated* serum osmolarity is greater than its molar concentration. The increased osmotic activity of ethanol is thought to be due to its binding to water molecules. This study is conducted to determine the true osmotic contribution of ethanol to serum osmolality.

**Methods:** Baseline serum osmolality and ethanol concentration were measured on each serum sample. Varying amounts of ethanol were added to aliquots of serum in which the baseline serum ethanol concentration was undetectable. Repeat serum osmolality and serum ethanol concentration were measured after addition of ethanol.

**Results:** The range of serum ethanol concentration was 27.3–429.8 mg/dL. The serum osmolal gap attributed solely to ethanol is calculated based on the difference between *measured* serum osmolality before and *measured* serum osmolality after addition of ethanol. Our results demonstrated that the contribution of ethanol to serum osmolality can be calculated by dividing the serum ethanol level in mg/dl by 4.6. In addition, the relationship between serum ethanol concentration and osmolal gap due to ethanol was assessed by linear regression analysis. Linear regression analysis relating the osmolal gap due to ethanol and ethanol concentration yielded the following equation: Osmolal Gap (mOsm/kg H_2_O) = 0.23 (Ethanol [mg/dL]) – 1.43.

**Conclusion:** The osmolal concentration of ethanol can be calculated based on its molar concentration. We found no evidence for ethanol binding to water molecules over the range of ethanol concentration in this study.

## Introduction

Historically, it has been assumed that the osmotic contribution of ethanol to serum osmolality can be calculated based on its molar concentration. The osmolal concentration of ethanol is thought to be equal to the serum ethanol level in mg/dl divided by 4.6 given that its molecular weight is 46 g/mol (1). However, recent studies suggested that the osmolal concentration of ethanol is greater than its molar concentration. Purssell et al. demonstrated that the osmolal concentration of ethanol is best estimated by dividing the ethanol concentration by 3.7 rather than 4.6, whereas Garrard et al. found that the conversion factor of ethanol is 4.0 (2, 3). There are, however, limitations inherent in these studies as the osmolal gap due to ethanol was determined by the difference in the *measured* serum osmolality with ethanol and the *calculated* serum osmolarity excluding ethanol. This approach is inaccurate since there is an expected osmolal gap between the *measured* serum osmolality and the *calculated* serum osmolarity even if the serum ethanol concentration were zero. Therefore, in determining the osmolal gap due *solely* to ethanol, one must calculate the difference in the *measured* serum osmolality prior to addition of ethanol and *measured* serum osmolality after addition of ethanol. This osmolal gap due solely to ethanol has not previously been reported in the literature.

## Methods

UCLA Institutional Review Board waived the requirement for ethical approval and written informed consent for participants in this study due to the used samples being de-identified and discarded from use for patient care only. This was carried out in accordance with the national legislation and institutional requirements. Thirty-three serum samples with undetectable ethanol level were selected for this study. Serum ethanol concentration was measured with the Roche Cobas 8000 (Roche Diagnostics, Indianapolis, Indiana) on each sample. Serum osmolality (mOsm/kg H_2_O) was measured using a freezing point depression method on the OsmoPRO (Advanced Instruments, Inc. Norwood, Massachusetts) in duplicate. Then 0.5 mL aliquot of each sample was spiked with varying amount of 200 proof ethanol (Gold Shield Dist. Inc. Hayward CA). Serum ethanol and osmolality were measured again with the same methods as described earlier. The average serum osmolality was determined from the duplicate results.

## Results

**Figure 1:**
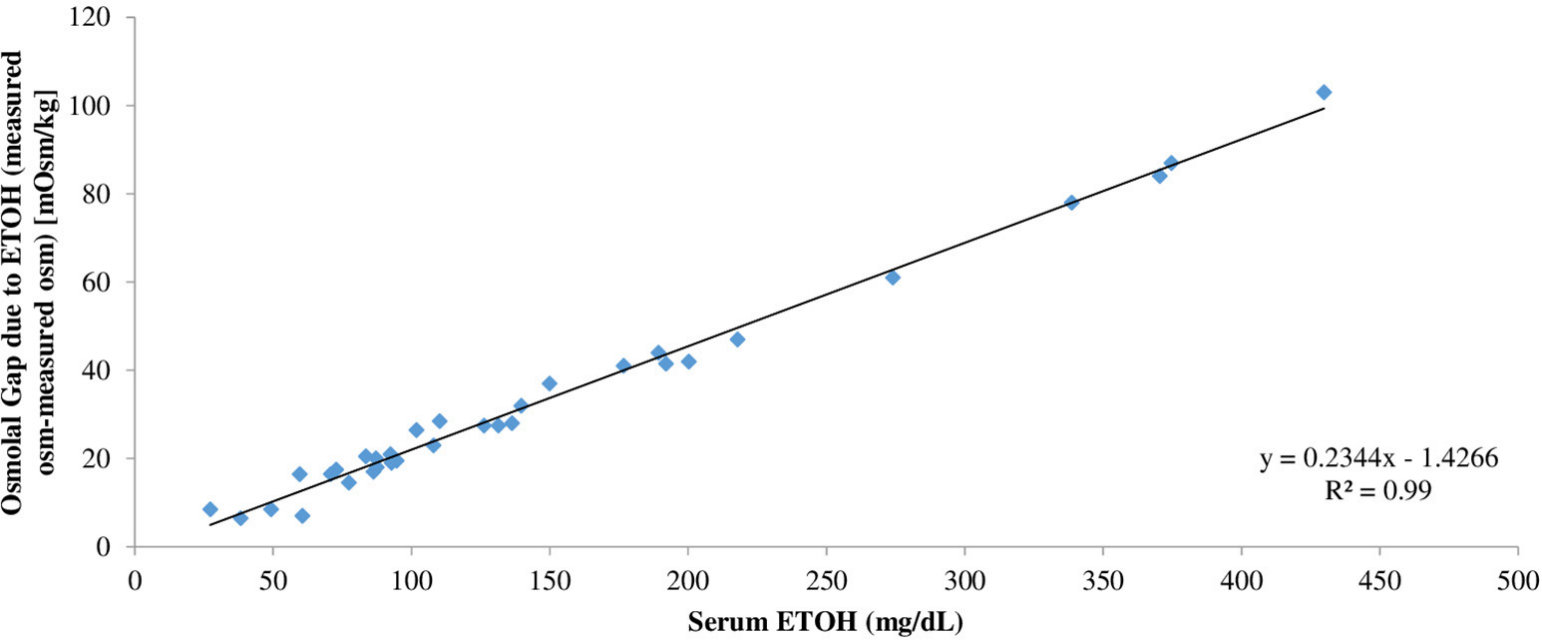
Linear regression analysis relating the osmolal gap *solely* due to ethanol based on the difference between *measured* serum osmolality after ethanol addition and *measured* serum osmolality before ethanol addition and serum ethanol concentration in mg/dL.

The range of the serum ethanol concentration was 27.3–429.8 mg/dL in samples spiked with ethanol (Table 1). The range of average serum osmolality was 277–307 mOsm/kg H_2_O in the original serum samples and 290.5–380 mOsm/kg H_2_O in samples spiked with ethanol (Table 1). The serum osmolal gap attributed *solely* to ethanol was calculated based on the difference between *measured* serum osmolality before and *measured* serum osmolality after addition of ethanol. To determine the conversion factor of ethanol from unit of mg/dL to unit of mOsm/kg H_2_O, the serum ethanol concentration in mg/dL on each serum sample was then divided by the serum osmolal gap attributed to ethanol for that sample. In contrast to the previous two studies by Purssell et al. and Garrard et al., the true average conversion factor of ethanol is 4.6 ± 0.16 since this calculation is performed using the serum osmolal gap *solely* attributed to ethanol based on the difference between *measured* serum osmolality before and *measured* serum osmolality after addition of ethanol (Table 1). In addition, linear regression analysis was performed to assess the relationship between the osmolal gap due to ethanol and serum ethanol concentration in mg/dL. Linear regression analysis relating the osmolal gap *solely* due to ethanol based on the difference between *measured* serum osmolality before and after ethanol addition and serum ethanol concentration yielded the following equation: Osmolal Gap (mOsm/kg H_2_O) = 0.234 (Ethanol [mg/dL]) – 1.427 (95% CI: slope 0.226–0.243, intercept −2.971 to 0.118) (Figure 1).

**Table 1:** Patient data.

	**Serum ETOH**	**Measured serum osmolality (no ETOH)**	**Measured serum osmolality (no ETOH) (Repeat)**	**Average measured serum osmolality (no ETOH)**	**Measured serum osmolality (with ETOH)**	**Measured serum osmolality (with ETOH) (Repeat)**	**Average measured serum osmolality (with ETOH)**	**Osmolal gap due to ETOH**	**Conversion factor for ETOH**
**Sample**	**mg/dL**	**mOsm/kg**	**mOsm/kg**	**mOsm/kg**	**mOsm/kg**	**mOsm/kg**	**mOsm/kg**	**mOsm/kg**	
1	374.6	293	293	293	379	381	380	87	4.31
2	86.2	296	296	296	314	312	313	17	5.07
3	192.0	301	299	300	343	340	341.5	41.5	4.63
4	110.1	296	299	297.5	326	326	326	28.5	3.86
5	83.5	291	289	290	312	309	310.5	20.5	4.07
6	217.9	294	292	293	343	337	340	47	4.64
7	274.0	290	289	289.5	353	348	350.5	61	4.49
8	70.8	298	300	299	315	316	315.5	16.5	4.29
9	126.2	293	295	294	322	321	321.5	27.5	4.59
10	189.2	295	298	296.5	341	340	340.5	44	4.30
11	92.8	283	282	282.5	301	302	301.5	19	4.88
12	429.8	283	283	283	385	387	386	103	4.17
13	87.5	282	277	279.5	300	295	297.5	18	4.86
14	338.6	301	300	300.5	380	377	378.5	78	4.34
15	77.3	283	283	283	299	296	297.5	14.5	5.33
16	92.4	292	291	291.5	312	313	312.5	21	4.40
17	370.5	293	291	292	374	378	376	84	4.41
18	49.2	296	295	295.5	306	302	304	8.5	5.79
19	87.1	285	282	283.5	303	304	303.5	20	4.36
20	107.9	283	283	283	309	303	306	23	4.69
21	60.6	281	286	283.5	287	294	290.5	7	8.66
22	149.9	294	293	293.5	330	331	330.5	37	4.05
23	72.8	297	297	297	315	314	314.5	17.5	4.16
24	200.2	283	284	283.5	326	325	325.5	42	4.77
25	176.6	287	290	288.5	330	329	329.5	41	4.31
26	38.3	285	290	287.5	293	295	294	6.5	5.89
27	101.8	286	286	286	313	312	312.5	26.5	3.84
28	94.6	293	294	293.5	313	313	313	19.5	4.85
29	131.4	287	287	287	313	316	314.5	27.5	4.78
30	27.3	297	297	297	304	307	305.5	8.5	3.21
31	136.3	307	307	307	336	334	335	28	4.87
32	59.6	278	276	277	295	292	293.5	16.5	3.61
33	139.6	292	293	292.5	325	324	324.5	32	4.36
Average	146.87	290.76	290.82	290.79	324.15	323.42	323.79	33.00	4.63
SEM	18.03	1.19	1.25	1.21	4.53	4.55	4.53	4.25	0.16

*ETOH, Ethanol*.

### Statistics

The data were presented as mean ± SEM. In addition, linear regression was performed to define the relationship between the osmolal gap *solely* due to ethanol and serum ethanol concentration.

## Discussion

The serum osmolal gap is an important clinical tool utilized in the evaluation of suspected toxic alcohol poisoning. An increased serum osmolal gap is suggestive of methanol or ethylene glycol intoxication. However, concomitant ethanol ingestion also contributes to the increased serum osmolal gap and must be accounted for in the evaluation of methanol or ethylene glycol intoxication. Historically, the osmolal concentration of ethanol is assumed to be equal to its molar concentration. Given that ethanol has a molecular weight of 46 g/mol, the osmolal concentration of ethanol is calculated as the serum ethanol concentration in mg/dL divided by 4.6 (1). However, recent data suggested that ethanol reduces the effective serum water volume (i.e., free solvent) and therefore contributes more osmoles per kg H_2_O than its molar concentration in a manner similar to that of sucrose. It has been demonstrated that the osmotic pressure exerted by sucrose was greater than that expected based on its molar concentration (4). When the water that bound with sucrose was deducted from the total water available in the system, the free-solvent model provided an accurate prediction of the osmotic pressure data. Indeed, by assuming that each sucrose molecule binds 4.2 water molecules, the calculated osmotic pressure was predictably accurate up to 190 atm (4). Purssell et al. demonstrated that the osmolal contribution of ethanol is best determined by dividing the serum ethanol concentration in mg/dL by 3.7 rather than dividing by 4.6 (2). On the other hand, Garrard et al. showed that the contribution of ethanol to the osmolal gap is calculated as the ethanol concentration in mg/dL divided by 4.0 rather than 4.6 (3). However, there is a significant limitation inherent in both of these studies. In these studies, the osmolal gap was determined by the difference in the *measured* serum osmolality with ethanol and *calculated* serum osmolarity excluding ethanol. The problem with the conversion factors derived from their studies is that they did not consider the contribution of the solutes present in the serum other than ethanol, sodium, urea nitrogen and glucose to serum osmolality, i.e., the expected osmolal gap when serum ethanol concentration is zero.

In our study, to determine the serum osmolal gap due *solely* to ethanol, the true serum osmolal gap due to ethanol was determined by the difference in the *measured* serum osmolality prior to addition of ethanol and the *measured* serum osmolality after addition of ethanol. Consequently, the true conversion factor of ethanol from unit of mg/dL to unit of mOsm/kg H_2_O was determined by dividing the serum ethanol concentration in mg/dL of each serum sample by the serum osmolal gap due solely to ethanol for that sample (Table 1). As shown in Table 1, the true average conversion factor of ethanol is 4.6 ± 0.16. Alternatively, the osmolal contribution due solely to ethanol can be predicted based on its concentration in mg/dL by the following linear regression equation: Osmolal Gap (mOsm/kg H_2_O) = 0.23 (Ethanol [mg/dL]) – 1.43 (Figure 1). Based on the results of our study, we demonstrated that an ethanol solution with concentration up to 430 mg/dL still behaves as if it is an ideal solution. Our data does not suggest that ethanol reduces the effective water volume in serum over the range of ethanol concentration in this study.

The differences between the two previous conversion factors of 3.7 and 4.0 and the true conversion factor of 4.6 are likely the result of variations in the expected serum osmolal gap between the *measured* serum osmolality with ethanol and *calculated* serum osmolarity excluding ethanol, i.e., variations in the concentration of non-ethanol solutes other than serum sodium, serum urea nitrogen and serum glucose. In Table 2, the predicted osmolal gap due to ethanol was calculated using the conversion factor of 3.7, 4.0, and 4.6, respectively. As shown in Table 2, the average difference between the calculated osmolal gap due to ethanol based on the conversion factor of 3.7 and calculated osmolal gap due to ethanol based on the true conversion factor of 4.6 was 7.77 ± 0.95. The average difference between the calculated osmolal gap due to ethanol based on the conversion factor of 4.0 and calculated osmolal gap due to ethanol based on the true conversion factor of 4.6 was 4.79 ± 0.59. As expected, the average differences between the calculated osmolal gap due to ethanol based on the incorrect conversion factors and the true conversion factor were less than the normal serum osmolal gap of 10 mOsm/kg H_2_O, i.e., the normal serum osmolal gap due to non-ethanol solutes other than serum sodium, serum urea nitrogen, and serum glucose (5).

**Table 2:** Comparison of osmolal gaps due to ethanol based on various conversion factors.

	**Serum ETOH**	**Serum ETOH/3.7**	**Serum ETOH/4**	**Serum ETOH/4.6**	**(Serum ETOH/3.7) – (Serum ETOH/4.6)**	**(Serum ETOH/4) – (Serum ETOH/4.6)**
**Sample**	**mg/dL**	**mOsm/kg**	**mOsm/kg**	**mOsm/kg**	**mOsm/kg**	**mOsm/kg**
1	374.60	101.24	93.65	81.43	19.81	12.22
2	86.20	23.30	21.55	18.74	4.56	2.81
3	192.00	51.89	48.00	41.74	10.15	6.26
4	110.10	29.76	27.53	23.93	5.82	3.59
5	83.50	22.57	20.88	18.15	4.42	2.72
6	217.90	58.89	54.48	47.37	11.52	7.11
7	274.00	74.05	68.50	59.57	14.49	8.93
8	70.80	19.14	17.70	15.39	3.74	2.31
9	126.20	34.11	31.55	27.43	6.67	4.12
10	189.20	51.14	47.30	41.13	10.00	6.17
11	92.80	25.08	23.20	20.17	4.91	3.03
12	429.80	116.16	107.45	93.43	22.73	14.02
13	87.50	23.65	21.88	19.02	4.63	2.85
14	338.60	91.51	84.65	73.61	17.90	11.04
15	77.30	20.89	19.33	16.80	4.09	2.52
16	92.40	24.97	23.10	20.09	4.89	3.01
17	370.50	100.14	92.63	80.54	19.59	12.08
18	49.20	13.30	12.30	10.70	2.60	1.60
19	87.10	23.54	21.78	18.93	4.61	2.84
20	107.90	29.16	26.98	23.46	5.71	3.52
21	60.60	16.38	15.15	13.17	3.20	1.98
22	149.90	40.51	37.48	32.59	7.93	4.89
23	72.80	19.68	18.20	15.83	3.85	2.37
24	200.20	54.11	50.05	43.52	10.59	6.53
25	176.60	47.73	44.15	38.39	9.34	5.76
26	38.30	10.35	9.58	8.33	2.03	1.25
27	101.80	27.51	25.45	22.13	5.38	3.32
28	94.60	25.57	23.65	20.57	5.00	3.08
29	131.40	35.51	32.85	28.57	6.95	4.28
30	27.30	7.38	6.83	5.93	1.44	0.89
31	136.30	36.84	34.08	29.63	7.21	4.44
32	59.60	16.11	14.90	12.96	3.15	1.94
33	139.60	37.73	34.90	30.35	7.38	4.55
Average	146.87	39.69	36.72	31.93	7.77	4.79
SEM	18.03	4.87	4.51	3.92	0.95	0.59

*ETOH, Ethanol*.

In another study by Khajuria et al. the serum ethanol concentration was measured in SI units, and the relationship between the osmolal concentration of ethanol and serum ethanol concentration in mmol/L was expressed by the following regression equation: Osmolal Gap = 1.2(Ethanol [mmol/L]) + 16.7 (6). In addition, Geller et al. demonstrated that the serum ethanol concentration in mmol/L is related to the osmolal gap by the formula: Ethanol [mmol/L] = 0.83 x osmolal gap (7). Similar to the previous studies by Purssell et al. and Garrard et al., an inherent limitation of these studies is that the osmotic contribution of ethanol is also determined by the difference in measured serum osmolality and calculated serum osmolality (6, 7).

Although our study is not a clinical prospective study, the true osmotic contribution of ethanol to serum osmolality is best determined by an *in-vitro* study. Alcoholic beverages that are safe for human consumption are <200 proof (100%) ethanol. In a clinical prospective study, consumption of ethanol <200 proof will result in a dilution of the osmolal concentrations of solutes in the serum due to the water content of the ingested alcoholic beverage. Therefore, changes in the measured serum osmolality will be due to both the ethanol content and water content of the ingested alcoholic beverage. In contrast, the addition of 200 proof (100%) ethanol to a serum sample *in-vitro* results in a change in measured serum osmolality that is due solely to the ethanol added.

In conclusion, we have demonstrated that the osmolal concentration of ethanol can be predicted based on its molar concentration and that the osmolal concentration of ethanol is equal to the serum ethanol level in mg/dl divided by 4.6. When evaluating patients with suspected methanol or ethylene glycol intoxication, this is the conversion factor that should be used clinically to determine the contribution of ethanol to the increased serum osmolal gap.

## Data Availability Statement

All datasets generated for this study are included in the article.

## Ethics Statement

UCLA Institutional Review Board has waived the requirement for ethical approval and written informed consent for participants in this study due to the samples used in this study being de-identified residual samples for patient care. This study was carried out in accordance with the national legislation and institutional requirements.

## Author Contributions

MN: conception and design of research. LS, LK, KT, MD, GR, and IK: conducted the experiments. MN, D-SN, and M-KN: analyzed the data. MN: interpreted results of experiments and drafted the manuscript. MN, LS, and IK: edited and revised the manuscript and approved final version of the manuscript. D-SN and M-KN: prepared figure and tables.

### Conflict of Interest

The authors declare that the research was conducted in the absence of any commercial or financial relationships that could be construed as a potential conflict of interest.
